# Interrater Reliability of Functional Movement Screening Test among Untrained Undergraduate Raters Undergoing a One-off Training Session

**DOI:** 10.21315/mjms-09-2024-740

**Published:** 2025-02-28

**Authors:** Zulezwan Ab Malik, Fadlin Sakina Abd Kadir, Engku Nurul Izzatul Iffah, Muhamad Hakimi M Yussof, M Nur Asraf Ismail, Nor Fazila Abd Malek, Ali Md Nadzalan, Hairul Anuar Hashim

**Affiliations:** 1 Universiti Pendidikan Sultan Idris, Tanjong Malim, Perak, Malaysia; 2 School of Health Sciences, Universiti Sains Malaysia, Health Campus, Kelantan, Malaysia

**Keywords:** FMS™, motor skills, reliability, rehabilitation

## Abstract

**Background:**

Functional Movement Screen (FMS)™ can be a valuable tool for assessing movement patterns and identifying potential movement dysfunctions. However, it is limited in terms of subjectivity and consistency of the ratings, especially among less proficient raters. Knowledge of minimally required training thresholds will provide valuable information on training adequacy. Thus, this study aimed to determine the interrater reliability of FMS™ among completely novice raters undergoing a one-off training session.

**Methods:**

Twenty active adults with no musculoskeletal injuries or muscular dysfunction performed seven FMS™ tasks while being recorded. Subsequently, 12 novice raters with no prior knowledge and skills about FMS™ rated the recorded movement videos at a normal play speed and without rewinding.

**Results:**

The interrater reliability analysis of the composite FMS™ score revealed an Intraclass Correlation Coefficient (ICC) of 0.60. Moreover, the mean coefficient for interrater reliability for the seven FMS™ components score is 0.35.

**Conclusion:**

The results implied that a one-off FMS™ training produced a large variability in the rating consistency, especially for rotary stability, deep squats, and in-line lunge ratings.

## Introduction

Injury prevention is an essential facet of sport and exercise participation. Injury may be prevented by identifying the risk factors that expose individuals and athletes to injuries. The risks can be identified via a movement screening process that could predispose individuals to injury. In this regard, the Functional Movement Screen (FMS™) plays a crucial role in assessing the presence of movement patterns that could impede performance or physical functions and increase risks of injury. Specifically, FMS™ can detect asymmetries and functional movement deficiencies, helping identify and mitigate potential risk factors. Indeed, mounting evidence suggests that more significant asymmetries in the body correspond to increased injury risk (1, 2).

Cook et al. (3, 4) proposed seven fundamental movement tasks, including deep squat, hurdle step, in-line lunge, shoulder mobility, active straight leg raises, trunk stability push-up, and rotary stability. A growing number of subsequent studies support the utility of these movement patterns in assessing functional movement deficiency and injury risk (e.g., 1, 2, 5). Thus, FMS™ may serve as a tool for swiftly identifying any movement asymmetries while concurrently informing their underlying causes. It can also be used as a baseline assessment for an individual’s physical capabilities, enabling related professionals to devise corrective interventions to help individuals achieve their movement objectives.

FMS™ movement tasks are scored using a 4-point ordinal scale (0–3) to obtain a total score of 0–21. It also includes three clearing tests: shoulder impingement, spinal extension, and spinal flexion tests to ascertain the presence of pain. The tests are interpreted with a score of “1” corresponding to the inability to perform the movement, “2” corresponding to performing the movement with compensation, and “3” corresponding to the ability to complete the movement without compensation correctly (3, 4, 6).

While FMS™ has been widely used and has practical significance, it does have several inherent limitations (7). Specifically, it has an inherent issue with scoring subjectivity, especially with multiple raters. The subjective assessment may introduce inconsistency and variability between evaluators, leading to potential discrepancies in results (8). This is especially true for novice assessors, whose lack of experience and training may give inconsistent and inaccurate scoring. Furthermore, novice raters may not be sufficiently familiar with movement assessment techniques or understand the criteria for scoring in the FMS™. Although the more training the raters have, the more proficient they will be, there are situations where minimally trained raters are needed. Thus, establishing the minimal training threshold may shed some light on the reliability of the rating.

Studies examining the rating consistency on the FMS™ have revealed some inconsistent findings concerning interrater and intrarater reliability among novice raters. For instance, Gribble et al. (9) examined intrarater reliability among three groups of raters with varied experience: raters who were certified athletic trainers with FMS™ experience, raters who were certified in Athletic Training (AT) but had no experience in FMS™, and undergraduate students without AT certification and FMS™ exposure. Their results revealed fair to strong intrarater reliability. Specifically, raters with at least one year of FMS™ experience had excellent intrarater reliability, followed by those with AT experience (0.758). Expectedly, raters without AT and FMS™ experience had the lowest intraters reliability (0.372).

Regarding interrater reliability, Gulgin and Hoogenboom (10) found excellent reliability (ICC = 0.88) between pairs of novice and experienced raters. In this study, the novice raters were those already certified in FMS™ scoring, making a comparison to other novice raters difficult. In another study, Leeder et al. (11) examined interrater reliability among experienced physiotherapists with no exposure to FMS™. They were only given instructions on how to score the recorded individual’s movements via a DVD and observed a good to excellent reliability coefficient (ICC: 0.9) (11). Similarly, Morgan et al. (12) found excellent interrater reliability among minimally trained raters. In Morgan et al.’s (12) study, each rater received a brief one-hour presentation on how the FMS™ is conducted and a two-hour lecture on the FMS™ in a class. They also had FMS™ scoring practice three times before the actual rating session. On the other hand, Shultz et al. (13) observed poor interrater reliability (0.38) among six raters with various backgrounds and clinical experience (one undergraduate student, one physical therapist, two athletic trainers, and two strength and conditioning coaches).

In summary, while the FMS™ can be a valuable tool for assessing movement patterns and identifying potential movement dysfunctions, it is limited by the subjectivity of the rating process, especially among less proficient raters. However, the extent of difference in ratings varies in different studies. Knowledge of minimally required training threshold will provide a starting point for the training plan. Thus, the primary objective of this study is to provide further evidence of the feasibility of administrating FMS™ among novice raters who underwent a one-off training session.

## Methods

This correlational study is designed to determine the interrater reliability of FMS™ among novice raters.

### Study Participants

Twenty active adults with no musculoskeletal injuries or dysfunction were recruited to perform seven FMS™ tasks. The inclusion criteria include an age range of 18 to 35, medically fit with no musculoskeletal injuries or dysfunction and consent to participate. The participants were required to perform seven FMS™ movement tasks: deep squat, hurdle step, in-line lunge, shoulder mobility, active straight leg raises, trunk stability push-up, and rotary stability. In addition, 12 novice undergraduate raters without any prior FMS™ skills and experience were included. Moreover, a Level 2-FMS™-certified and experienced rater was also included as a criterion for the rating.

### Procedure

Prior to the start of the study, relevant and approval were obtained. Ethical approval was obtained from the Human Research Ethics Committee board of the first author’s institution (Ref: 2021-0173-01). Furthermore, the participants provided signed consent to participate in this study. The participants performed seven FMS™ movements following a video recording based on the video provided by the researchers. The movements were repeated three times based on the protocol by Cook et al. (3, 4). They were recorded from frontal and sagittal views, and no coaching was allowed to the participants during the recording. Both right and left side views were recorded.

Subsequently, all raters were provided with a recorded video of the seven FMS™ movement tasks. The videos also included introductory information about FMS™, the clearing test, and the scoring criteria for the seven FMS™ movement tests.

### Rating Session

Prior to movement rating, a familiarisation session was conducted among the raters. Each rater received a one-hour presentation of the FMS™, including the seven movements and the scoring criteria. The raters also performed one session of FMS™ scoring practice session using two movement recordings.

Following the familiarisation session, the raters rated the pre-recorded movement at a normal play speed. No slow-motion viewing attempts were made to replicate the real-time scoring. The FMS™ standard scoring criteria for the seven screening tests were used. All scores from the raters were recorded on a data collection sheet.

### Statistical Analysis

The Statistical Package for the Social Science (SPSS) version 26.0 for Windows was used to analyse the data. The Intraclass Correlation Coefficient (ICC) was computed to establish the interrater reliability for the FMS™ composite score. Meanwhile, the unweighted Kappa statistic was used to establish the interrater reliability measurement for each rater. The value of Kappa over 80% is considered excellent agreement, above 60% is a substantial level of agreement, a rating between 40%–60% is considered a moderate agreement, and below 40% is considered poor to fair agreement (14). The level of significance was set at *p* ≤ 0.05. Fisher’s exact test was also used to compare each rater with the expert rater.

## Results

Twenty participants who performed the seven movement tasks had an average age of 22.80 ± 0.89, weight and height of 58.90 ± 8.42, and 164.25 ± 8.71, respectively. The results revealed a mean interrater reliability coefficient of 0.60 of the FMS™ composite score, suggesting moderate rating agreement.

Furthermore, criterion validity was established via correlation analysis between novice and expert ratings. As shown in Table 1, the results revealed an acceptable agreement between novice and expert scores. Overall, the mean correlation coefficient is 0.35, indicating a fair reliability coefficient.

Table 2 shows the interrater reliability results for the FMS™ components score, which ranges from none to slight, fair, and moderate agreement between raters. Rotary stability has the least consistent movement test ratings (kw = 0.073), whereas active straight leg raise has the most consistent ratings (kw = 0.450).

## Discussion

This study investigated the interrater reliability and validity of FMS™ scoring by novice undergraduate student raters who underwent a one-hour training session. The findings revealed a moderate level of reliability for the FMS™ composite score, with an average ICC of 0.60. These results contradict previous studies, such as those by Gulgin and Hoogenboom (10), Leeder et al. (11), Morgan et al. (12), Onate et al. (15) and Minick et al. (16) involving untrained FMS™ raters, which reported excellent interrater agreement. These contradictory findings may be partially attributed to the raters’ backgrounds. Specifically, the terms novice and untrained raters are used differently in different studies.

For instance, in Gulgin and Hoogenboom’s (10) study, their raters, although labelled novice, had received FMS™ certification, though with limited practice experience. In Onate et al.’s (15) study, their novice FMS™ raters were certified Strength and Conditioning Specialists with three years of practice experience. Moreover, in the Minick et al. (16) study, novice individuals were defined as having taken the standardised introductory training course and having used the FMS™ less than a year. Whereas, in Morgan et al. (12) study, although the raters received minimal training and were not FMS™ certified, they were halfway through their second year in the Doctor of Physical Therapy Programme. Raters in the present study were undergraduate sports and rehabilitation sciences students with limited clinical experience. We observed inconsistent ratings with two novice raters demonstrating moderate to excellent interrater reliability with an expert rater and one rater with particularly low reliability. The remaining raters exhibited low to fair reliability.

One notable point that may differentiate the rating consistency, despite not having the FMS™ certification and experience, is the movement science knowledge. Specifically, individuals with more excellent movement knowledge may require lesser training to rate the movement in the FMS™ test, as in Morgan et al.’s (12) study. Thus, more training is required for those without or with less movement science knowledge.

Our reliability results for FMS™ components ranged from “none to slight” to “moderate”. The most reliable score is the active straight leg raises rating with moderate reliability. A possible explanation is the relative ease of this movement and its scoring. On the other hand, three tests demonstrated the lowest interrater reliability: rotary stability, deep squat, and in-line lunges, which are consistent with Minick et al. (12), Schneiders et al. (17) and Teyhen et al. (18), who also reported minimal agreement between raters for rotary stability and deep squats. This pattern suggests that the assessments are particularly challenging to rate consistently, likely due to the involvement of multiple joints and segments in performing those tasks. The raters’ experiences and lack of clearly defined scoring criteria, especially midrange performance, may contribute to the rating consistency.

## Conclusions

In conclusion, our study pointed out two important conclusions: the training threshold depends on the knowledge of the raters regarding movement sciences. Specifically, individuals with sufficient movement science knowledge may require less training than those without or with lesser knowledge in movement science and related fields. Secondly, some test components may require a better understanding and more practice to be rated more consistently and accurately.

## Figures and Tables

**Table 1:** Inter-rater reliability of FMS™ composite scores between novice and expert raters

Raters	ICC	Interpretation (19)	95% Confidence Interval
R1	−0.25	Poor	−10.75–0.80
R2	0.36	Fair	−2.74–0.89
R3	0.86	Excellent	0.09–0.98
R4	−0.02	Poor	−4.96–0.82
R5	0.36	Fair	−0.85–0.87
R6	0.75	Good	−0.46–0.96
R7	0.29	Fair	−3.13–0.88
R8	0.57	Moderate	−1.49–0.93
R9	0.37	Fair	−2.66–0.89
R10	0.15	Poor	−3.92–0.86
R11	0.73	Good	−0.60–0.95
R12	0.36	Fair	−2.73–0.89

Mean	0.35	Fair	−2.79–0.89

**Table 2:** The Inter-rater reliability of FMS™ components scores

	Kappa (kw)	Interpretation (14)
Deep squat	0.157	None to slight
Hurdle step	0.217	Fair
In-line lunge	0.163	None to slight
Shoulder mobility	0.254	Fair
Active straight leg raise	0.450	Moderate
Trunk stability push-up	0.282	Fair
Rotary stability	0.073	None to slight
